# Microplastics and human health: unveiling the gut microbiome disruption and chronic disease risks

**DOI:** 10.3389/fcimb.2024.1492759

**Published:** 2024-11-25

**Authors:** Sudipta Sankar Bora, Rahul Gogoi, Madhurjya Ranjan Sharma, Anshu, Madhurjya Protim Borah, Priyadarshini Deka, Jitul Bora, Romen Singh Naorem, Jugabrata Das, Anju Barhai Teli

**Affiliations:** ^1^ Multidisciplinary Research Unit, Jorhat Medical College and Hospital, Jorhat, Assam, India; ^2^ Department of Agricultural Biotechnology, Assam Agricultural University, Jorhat, Assam, India; ^3^ Department of Medical Oncology, All India Institute of Medical Sciences, New Delhi, India; ^4^ Department of Biosciences and Bioengineering, Indian Institute of Technology Jammu, Jammu, India; ^5^ College of Horticulture and Farming System Research, Assam Agricultural University, Nalbari, Assam, India; ^6^ Department of Biochemistry, Jorhat Medical College and Hospital, Jorhat, Assam, India

**Keywords:** microplastic, human health, gut microbiota, dysbiosis, chronic diseases, inflammation

## Abstract

Microplastics (MPs), defined as plastic particles smaller than 5 mm, are increasingly recognized as environmental contaminants with potential health risks. These emerge as breakdown products of larger plastics and are omnipresent in marine, freshwater, and terrestrial ecosystems. They are primarily composed of polymers such as polyethylene, polypropylene, polystyrene, and additives that enhance their performance. MPs also adsorb harmful environmental chemicals like persistent organic pollutants and heavy metals, posing risks to human and environmental health. Human exposure to MPs occurs mainly through ingestion and inhalation, with MPs detected in food products, water, and even the air. MPs have been shown to accumulate in the gastrointestinal tract, disrupting the gut microbiome, and causing dysbiosis-a harmful imbalance between beneficial and harmful bacteria. This disruption has been linked to various health issues, including gastrointestinal disorders, systemic inflammation, and chronic diseases. Furthermore, the gut-brain axis may be affected, with potential neuroinflammatory consequences. As research continues to unravel the health impacts of MP exposure, understanding the mechanisms of accumulation and the broader implications on human health is crucial. This review highlights the effects of MPs on human health, emphasizing their impact on the gut microbiome. We discuss the potential connections between MP exposure and cardiometabolic and inflammatory diseases, and disorders related to the Gut-Brain Axis. By synthesizing the latest research, this work sheds light on the silent yet pervasive threat posed by MPs and underscores the importance of further studies to understand their health impacts fully.

## 1 Introduction

Plastics have become a crucial component of modern society, with global production reaching to 320 million tonnes annually. Plastic is a significant environmental contaminant due to its durability and toughness, surviving a lifetime in the environment (Okoffo et al., 2021). Its persistent nature leads to rapid accumulation and slow degradation, causing environmental issues like garbage buildup in landfills and water bodies. Every year, approximately eight million tonnes of plastic debris find their way into the ocean. Once in the environment, plastics undergo degradation due to factors such as physicochemical activity, UV radiation, and microbial action, breaking down into micro- and nanosized particles that further contribute to environmental contamination (Lamichhane et al., 2023). Larger plastic objects can breakdown into highly hazardous microplastic (MP) particles, which pose a significant risk to human health and the environment. Any plastic particle less than 5 mm to 1 µm along its longest dimension is referred to as microplastic. MPs have become a prevalent contaminant, found in various environmental conditions, including marine waters, freshwater bodies, wastewater, food, and even the air. Their presence has been sensed and detected globally at varying concentrations. Therefore, researchers are closely monitoring the global prevalence of MPs due to their environmental impact (Campanale et al., 2020).

The MP particles frequently detected in the environment are mainly composed of polymers such as polyethylene (PE), polypropylene (PP), polyvinyl chloride (PVC), polystyrene (PS), polyurethane (PU), and polyethylene terephthalate (PET). These materials are widely used in several consumer products. MPs may contain two types of chemicals: (A) additives and polymeric raw materials (e.g., monomers or oligomers) derived from the plastics: additives are substances that are purposefully added to plastic during the manufacturing process to give the material characteristics like color and transparency. They also improve the performance of plastic products by increasing their resistance to ozone, temperature, light radiation, mold, bacteria, and humidity as well as their mechanical, thermal, and electrical strengths (Maddela et al., 2023). (B) Chemicals absorbed from the environment: MPs can act as vectors for harmful chemicals and pathogens, adsorbing environmental pollutants like persistent organic pollutants (POPs) and heavy metals, causing toxicological effects upon ingestion. Some newly introduced bio-based plastics, like polylactic acid (PLA), and biodegradable plastics, such as oxo-degradable polyolefins, can also be found as MPs in the environment. The degradation of these MPs is often incomplete under natural environmental conditions, leading to the persistence of small plastic particles. MPs are also introduced into the environment through several other pathways, including atmospheric deposition, land-based sources, fertilizers, artificial turf, road runoff, landfills, air transportation, etc. (Ainali et al., 2022).

A study examined the impact of MPs and deltamethrin on the microbiota of a three-level food chain (daphnids, damselflies, and dragonflies), revealing that exposure to MPs and pollutants affects microbiomes at higher levels, potentially through direct transfer or predation (Varg et al., 2022). As such, the omnipresence of MPs in the environment has raised serious concerns about their long-term effects on human health particularly their impact on the gastrointestinal (GI) tract, the inhabiting gut microbiome, and their potential link to chronic diseases. Humans get exposed to MPs mainly through ingestion and inhalation. MPs have been found in various food products such as seafood, table salt, water (tap and bottled), and even honey. Seafood consumption significantly exposes humans to MPs, which accumulate in marine organisms’ tissues and eventually enter the human diet. The inhalation of urban air also contributes to MP exposure in the human (Li et al., 2024).

MPs can accumulate in the GI tract after being ingested and persist there for a long time due to their resistance to digestion. Multiple studies in animal models have reported the physical interaction of MPs with the gut lining leading to mechanical injuries and potentially evoking inflammatory responses. Additionally, MPs can increase intestinal permeability, a condition known as ‘leaky gut’, allowing harmful particles to enter the bloodstream (Turroni et al., 2021). The gut microbiome (nicknamed as ‘second genome’), a complex and dynamic community of microorganisms that play a critical role in digestion and metabolism, immune function, and overall well-being, is particularly vulnerable to MP exposure. Multiple studies have linked MP exposure to dysbiosis, a harmful imbalance between beneficial and pathogenic bacteria in the gut. Microbial dysbiosis can lead to impaired gut function, weakened immunity, and increased risk of GI disorders (Fackelmann and Sommer, 2019). Furthermore, MP-induced modulations in the gut microbiome can result in systemic inflammation, a known risk factor for a range of chronic diseases. Beyond the gut, the health impacts of MP exposure extend to systemic inflammation, which can lead to other chronic conditions such as obesity, diabetes, cardiovascular diseases, and autoimmune disorders. MPs are also increasingly linked to neuroinflammation, potentially affecting the Gut-Brain Axis and leading to neurological and psychological disorders (Sofield et al., 2024).

While our current understanding of MP-related health issues is still emerging, it is evident that these particles pose a silent yet significant risk. Continued rigorous research is needed to fully elucidate the mechanisms by which MPs impact human health and to assess the long-term consequences of exposure. The accumulation of MPs in the GI tract, dysbiosis of the gut microbiome, and systemic inflammation highlight the need for further research and policy interventions. This review focuses on various sources and prevalence of MPs in the environment, pathways, and mechanisms of MP exposure, accumulation in the GI tract, and impact on the gut microbiome. It further highlights the potential health implications (immune disorders, hepatic and renal health, cardiometabolic diseases, and Gut-Brain-Axis consequences) raising awareness about the risks associated with MP exposure.

## 2 Sources of microplastics

MPs are often derived from two principal sources-the primary source and the secondary source that produce various-sized plastic particles in the environment. Plastic pellets, microbead-containing personal care items, paint, washing wastewater, sewage sludge, artificial grass, rubber roads in cities, and automobile tires are the primary sources of environmental MPs. Secondary sources include plastic bags and bottles, fishing ware, farming film, and other large-scale plastic wastes (An et al., 2020) (**Table 1**). Because the number of vehicles on the road is increasing at a rapid pace worldwide, vehicle tires are considered one of the most significant sources of environmental MPs among these sources (Kole et al., 2017). Primary MPs are purposefully manufactured for specific applications, which include cosmetic abrasives, drug vectors, and industrial and engineering applications such as air blasting. These MPs are usually difficult to remove using sewage disposal technologies, and once they enter wastewater, they will ultimately accumulate in the environment. Secondary MPs originate from larger plastics as they are progressively fragmented into smaller pieces by multiple, complex environmental conditions such as wind, waves, temperature, and UV light (Ziajahromi et al., 2017).

**Table 1:** Various sources, composition, and conformation of microplastics.

Sources	Composition and structure	Shape	Size	Reference
Shower gels	Polyethylene	Irregular shapes	422 ± 185 μm	(Bashir et al., 2021)
Facial cleansers	Polyethylene	Spherical and irregular shapes	Higher than 0.5 mm	(Fendall and Sewell, 2009)
Car tires	Polypropylene/acrylic/nylon/rubber	Fragment/fiber	Higher than 500 μm	(Tamis et al., 2021)
Beverage products	Polyamide/acrylonitrile–butadiene–styrene/poly(ester-amide)/poly(ethylene terephthalate)	Fibers/fragments	0.1-3.0 mm	(Jin et al., 2021)
Facial scrubs	Facial scrubs	Facial scrubs	Facial scrubs	(Zhou et al., 2023)
Textile industrial area	Polyester	Fiber	0.1-1.0 mm	(Balasaraswathi and Rathinamoorthy, 2022; Periyasamy and Tehrani-Bagha, 2022)
Cosmetic products	Polyethylene	Irregular/granular/spherical	54-115 μm	(Bashir et al., 2021; Cubas et al., 2022)
Plastic mulch	Polyester, polypropylene	Fiber/fragment/foam/film	Higher than 500 μm	(Khalid et al., 2023)
Industrial sources	Polyethylene/nylon/polypropylene	Films/fragments/lines/granules/sheets/lines	0.5-1.0 mm	(Long et al., 2021; Osman et al., 2023)
Mariculture activities	Polyester/polypropylene/polyethylene/polyamide (nylon)/polystyrene/polyoxymethylene/polyetherurethane/polybutylene terephthalate	Fragments/flakes/fiber/foam	Less than 0.25 mm	(Chen et al., 2018; Xu et al., 2024)
Anthropogenic activity	Polystyrene/polyethylene/polypropylene	Fiber/styrofoam/fragment/film/pellet	Less than 0.5 mm	(Lin et al., 2022; Han et al., 2023)
Fishing and shipping activities	Ionomer surlyn/acrylic (acryl fiber)/polyetherimide/polyphenylene sulphide/ethylene vinyl alcohol/acrylonitrile/nylon/polyisoprene/polyvinyl chloride/ethylene–vinyl acetate/polyurethane	Fiber/pellet/fragment	1489 ± 1017 μm	(Wright et al., 2021; Alberghini et al., 2022)
Urban sewage	Polyethylene/polystyrene/polypropylene	Fragment/lines/foam/film	1.0-4.75 mm	(Dalu et al., 2021; Zhang et al., 2022)
Domestic, agriculture effluent, industry, upstream inflow, and airborne settlement	Polyethylene terephthalate/polyethylene/polypropylene/polystyrene/polycarbonate/polyvinyl chloride/cellulose propionate/polyamide/ethylene–vinyl acetate copolymer	Pellets/fragments	0.05-5.0 mm	(Osman et al., 2023)
Local inputs/ocean transport	Polypropylene/polyester/polyester/polyethylene	Fiber/flake/film/granule	2.0-2.5 mm	(Li et al., 2020; Long et al., 2022)
Artificial ecosystems	Polyethylene/rayon/polypropylene	Fiber/flake/film/granule	Less than 1.0 mm	(Pandey et al., 2022)

## 3 Prevalence in the environment and human exposure

Exposure routes of MPs to humans include ingestion, inhalation, and dermal penetration, with ingestion being the primary route. Due to the high concentration of MPs in the ocean, reaching up to 102,000 particles per cubic meter, seafood is considered one of the main sources of MPs through ingestion. In the case of inhalation-mediated exposure, synthetic textiles, and city dust are regarded as the most significant sources of primary MPs. Plastic fragments shed from clothes, furniture, textiles, and building materials contribute to secondary exposure outside. Plastic fibers, commonly found in carpets, sofas, and chairs at concentrations of 19.6 fibers per cubic meter, are responsible for indoor exposure (Sun and Wang, 2023). Dermal absorption of MPs can occur through the use of personal care products such as hand cleansers, body scrubs, face masks, toothpaste, etc. Human skin, with a surface area of approximately 1.5-2.0 square meters, serves as a critical interface with the environment, exposing it to the ubiquitous presence of MPs. This extensive surface area increases the potential for dermal contact with MPs. **Table 2** summarizes the prevalence and sources of various MPs with their size and concentration.

**Table 2:** Prevalence of microplastic in various sources, their compositional types, size, and concentration.

Prevalence	Source	Polymer type	Size	Concentration	Reference
Air	ambient air (outside)	polyethylene terephthalate, polyethylene, polypropylene	0.004-3 mm	between <1 to >1000 MPs/m^3^	(O’Brien et al., 2023)
	ambient air (inside)		0.004-0.398 mm	<1 MPs/m^3^ to 1583 ± 1181 MPs/m^3^	(O’Brien et al., 2023)
	rural farmland	polyester, nylon, polyolefin, PTFE, PE	<0.2-4.2 mm	137 ± 57 MPs/m^3^	(Kannan and Vimalkumar, 2021)
	Wetland	polyvinyl chloride, polyethylene, polypropylene	0.005-1 mm	97 ± 33 MPs/m^3^	(Kannan and Vimalkumar, 2021)
	Mountain		2 mm-50 mm	70 ± 18 MPs/m^3^	(Kannan and Vimalkumar, 2021)
Dust	Deposition concentrations (outdoor)	polyethylene, polystyrene, polypropylene, polyethylene terephthalate	2 mm-50 mm	0.5 and 1357 MPs/m^2^/day	(O’Brien et al., 2023)
	Deposition concentrations (indoor)		<2 mm	475 to 19,600 MPs/m^2^/day	(O’Brien et al., 2023)
	Road dust	polyvinyl chloride, polyethylene, polypropylene	2 mm-50 mm	477 MPs/g	(O’Brien et al., 2023)
	Snow	polyethylene terephthalate, polyethylene, polypropylene	2 mm-50 mm	between 0.1 and 30,000 MPs/L	(O’Brien et al., 2023)
	urban transit station	Cotton, polyester, nylon, polyolefin, PTFE, PE	2 mm-50 mm	287 ± 72 MPs/m^3^	(Kannan and Vimalkumar, 2021)
Water	Fresh water and drinking water	PE, PP, PS, PVC, PET	0.1-5.0 mm	1 × 10^−2^ to 10^8^/m^3^	(Neelavannan and Sen, 2023)
	Wastewater		10-5000 µm	1,000 to 100,000 particles per liter	(Neelavannan and Sen, 2023)
	Lake water	PP, PS, PE	0.1-5.0 mm	21 ± 13 particles/L	(Ajay et al., 2021)
	Groundwater		0.1-5.0 mm	3-23 items/L	(Kannan and Vimalkumar, 2021)
	Bore and well water	PA, PE, PE	0.1-5.0 mm	Mean 4.2 particles/L	(Selvam et al., 2021)
Food and beverages	Table salt	PET, PP, PE	100-5000 mm	5400 particles per kilogram	(Kwon et al., 2020)
	Fish	EVA, EPDM, PVF, PS, PTFE, PET, PP	760-6,000 µ	103 ± 41 to 183 ± 51 particles/fish	(Rubio-Armendáriz et al., 2022)
	Beer and soft drinks	PP, PE, polyacrylamide	Not specified	8-117 particles/L	(Diaz-Basantes et al., 2020)
	Soft and energy drinks, beer, cold tea	PET, PA, polyester, acrylonitrile-butadiene-styrene	25 µm	6-28 particles/L	(Smith et al., 2018)
	Bottled mineral water	PP, PE, PET, PS, PA, polyester	>100 µm	3.16 × 10^7^-1.1 × 10^8^ particles/L	(Zuccarello et al., 2019)
Human	Human placenta	PP, PET	5-10 µm	12 fragments in 4 placentas	(Ragusa et al., 2021)
	Lung tissue	PP, PE, PVC, cellulose acetate, polyamide, PS, PU	<5.5 µm	70 particles per lung	(Amato-Lourenço et al., 2021)

### 3.1 Mechanisms of microplastic ingestion

A 2016 UN report identified over 800 species of animals contaminated by plastics, marking a 69% increase compared to the 247 species listed in a 1977 study (Murray and Cowie, 2011). Of these 800 species, 220 were found to ingest MPs debris in nature. MPs have been found in different foods such as fish and seafood, table salt, beer, honey and sugar, etc. Soil contamination from discarded packaging or plastic agricultural equipment results in the presence of this substance (Wang et al., 2019). Studies show that humans eat at least 50,000 microplastic particles annually because of the infiltrated food chain, drinking water, and breathing air. MPs can be consumed from seven food sources: bottled water, beer, seafood (shellfish and fish), salt, tea bags, canned food, and ready meals. Water in plastic bottles that are used for drinking is one of the major sources, which may result in ingesting around 130,000 fragments of MP in the human body annually. The infiltration only gets worse when the bottle is exposed to direct sunlight. The tap water contains tiny plastic bits, but the level in bottled water is double that of tap water. The presence and persistence of MPs in numerous shellfish species across various global regions have been frequently observed, including clams, mussels, oysters, scallops, winkles, etc (Li et al., 2021). Tiny plastic fibers are present in their entire body including bivalves, which are consumed by humans. In addition, one kilogram of sea salt contains 212 particles of MP. The chemical Bisphenol A, or BPA, used to harden plastic, is the major health risk associated with canned foods as it leaks into the food within cans. Finally, ready-to-eat meals usually served in plastic containers add more MPs to the human diet (Praveena and Laohaprapanon, 2021).

**Figure 1:**
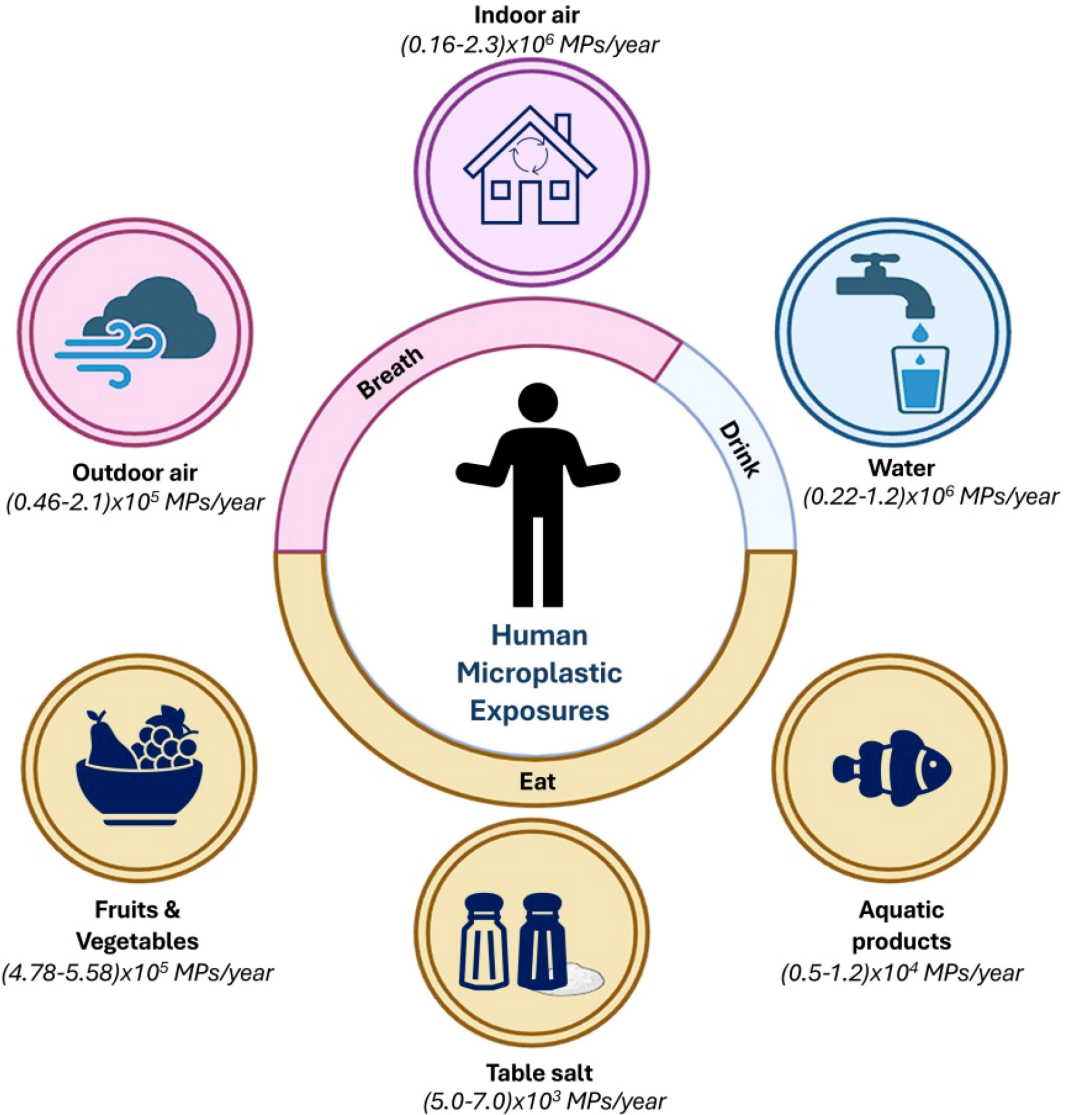
Representative schematic showing the annual intake of microplastics (MPs) by humans through various routes, categorized into breathing (airborne exposure) and eating/drinking (dietary exposure). It gives a comprehensive overview of how different environmental sources contribute to the ingestion or inhalation of MPs yearly (Yang et al., 2023). It shows the pervasive presence of MPs in the environment and their inevitable incorporation into human life through daily activities.

MPs pose a potential threat to human health due to their common presence in daily necessities. Therefore, it is important to understand the pathways of human exposure to MPs which include ingestion, inhalation, and skin contact as the common ways (**Figure 1**). Among these pathways, ingestion is the major exposure route (Prata et al., 2020). People are often exposed to MPs in multiple ways simultaneously.

A. Ingestion:

1. Seafood: Seafood provides almost 3 billion people worldwide with approximately 20% of their protein intake, making it the most important food commodity consumed globally and ultimately a plausible route for MPs leaching into the human body (FAO, 2024). MPs are found in many species intended for human consumption, including invertebrates, crustaceans, and fish. Several marine organisms, especially filter feeders such as oysters, mussels, and shellfish, can channel MPs to the human body. Plastic particles are often found concentrated in the digestive tracts of marine and freshwater organisms, such that bivalves and small fish consumed whole are more likely to expose MPs to the human diet (Li et al., 2015). Europeans are exposed to about 11,000 particles/person/year of MPs due to shellfish consumption (Van Cauwenberghe and Janssen, 2014). Food consumption mediated intake of plastic particles in the human body is estimated to be 39,000-52,000 particles/person/year (Cox et al., 2019).

2. Fish: Globally, fish provide approximately 4.3 billion people with 15 percent of their animal protein intake (FAO, 2024). Importantly, the ingestion of MPs by fish *in situ* has been widely reported, including by commercial species, although the quantity of ingested MPs is low. The occurrence of MPs in the GI tract of fish does not provide direct evidence for human exposure, as this organ is usually not consumed (Zazouli et al., 2022).

3. Salt: Sea salt has been found to contain MPs and may contribute to the potential long-term adverse effects resulting from human exposure to these particles (Peixoto et al., 2019). Salt is mostly produced by the distillation of seawater, which contains MP particles. It is difficult to avoid MPs in final sea salt products without further purification steps.

4. Other Foods: MPs have also been detected in other foods, such as honey, table sugar, fruits, and vegetables, likely due to environmental contamination during processing or packaging.

5. Water: Most of the urban water sources are found to be polluted with MPs due to wastewater and landfill leachate discharge. Additionally, studies have suggested the presence of MPs in both tap water and bottled water. These MPs can originate from the breakdown of plastic pipes, the packaging process, or from the environment (Chu et al., 2022). Drinks like beer and soft drinks have also been found to contain MPs, likely due to contamination during production, processing, or from the water used (Pironti et al., 2021).

B. Inhalation: Major sub-classifications of airborne MPs are indoor air, outdoor air, dust, and occupational/industrial exposure. Primary MPs found in the air are PE, PS, and PET particles and fibers with size ranges of 10-8000 μm (Kumar et al., 2022). Household dust often contains MPs, which can become airborne and inhaled during activities like cleaning or when disturbed by movement within the home. Similarly, workers involved in industries related to plastic production, recycling, or the handling of synthetic materials may be exposed to higher levels of airborne MPs, leading to increased inhalation risks (Zhao et al., 2023b). Indoor airborne MPs arise from synthetic textiles, carpets, and household dust. Inhalation of these particles can occur during regular breathing, especially in poorly ventilated spaces. Outdoor air has high concentrations of MPs originating from outdoor sources such as car tire wear, construction materials, and the degradation of plastics in the environment. These particles can be inhaled by individuals living in or passing through these areas. The largest source of MPs (84%) in the atmosphere comes from the road (Brahney et al., 2021). It is reported that the median concentration of MP fibers is 5.4 fibers/m^3^ in the outdoor air and 0.9 fibers/m^3^ in the indoor air in Paris (Jin et al., 2017). The average concentration of MPs is 1.42 particles/m^3^ in the outdoor air in Shanghai, and the size range is 23-5000 μm (Liu et al., 2019). It is estimated that annual MP consumption ranges from 74,000 and 121,000 particles when both oral intake and inhalation are considered (Cox et al., 2019). A study has detected MP particles smaller than 5.5 μm and MP fibers with the size of 8.12-16.8 μm in human lungs, whose main components were PE and PP (Amato-Lourenço et al., 2021). The size of MPs detected in lung tissue is smaller than that in the atmosphere. This further confirms that humans can be exposed to MPs by inhalation and prompts attention to the potential harm to the human body.

C. Skin Contact

MPs are usually considered not to pass through the skin barrier, but they can still increase exposure risk by depositing on the skin. For example, the use of consumer products containing MPs (such as face cream and facial cleanser) will increase the exposure risk of PE (Hernandez et al., 2017).

1. Dermal Contact:

• Lifestyle industry

Some cosmetics, such as exfoliating scrubs, contain microbeads, which are further leached to the environment. These MPs can come into contact with the skin and potentially be absorbed, though the extent of dermal absorption is not fully understood. Synthetic fibers from clothing can shed MPs, which may come into contact with the skin. Repeated or prolonged exposure may lead to potential absorption through the skin (Samanta et al., 2022).

• Contact with Contaminated Water

MPs present in tap water can come into contact with the skin during bathing or showering. Although the skin acts as a barrier, certain MPs could potentially penetrate the skin, especially if they are small enough. Swimming in contaminated water bodies, such as oceans, lakes, or rivers, may expose the skin to MPs present in the water. Protective mobile phone cases (PsMPCs) can generate MPs during use, which are transferred to human hands. When children crawl or play, they may come into contact with MPs on the ground. During the dermal exposure of MPs, some typical plastic additives, including brominated flame retardants (BFRs), bisphenols (BPs), triclosan (TCS), and phthalates may be absorbed (Wu et al., 2022).

## 4 Impact of microplastics on human health

The gut microbiota is central to energy homeostasis and nutrient metabolism, and disruptions caused by MPs can lead to imbalances in energy storage and expenditure (Fujisaka et al., 2023). The inflammatory responses triggered by MPs can interfere with insulin signaling pathways. Chronic low-grade inflammation, a hallmark of obesity, further exacerbates insulin resistance, a key feature of type 2 diabetes (Rohm et al., 2022). Thus, MP exposure indirectly contributes to these metabolic disorders by fostering an inflammatory environment and disrupting metabolic regulation.

### 4.1 Impact of microplastics on the gut microbiome

**Figure 2:**
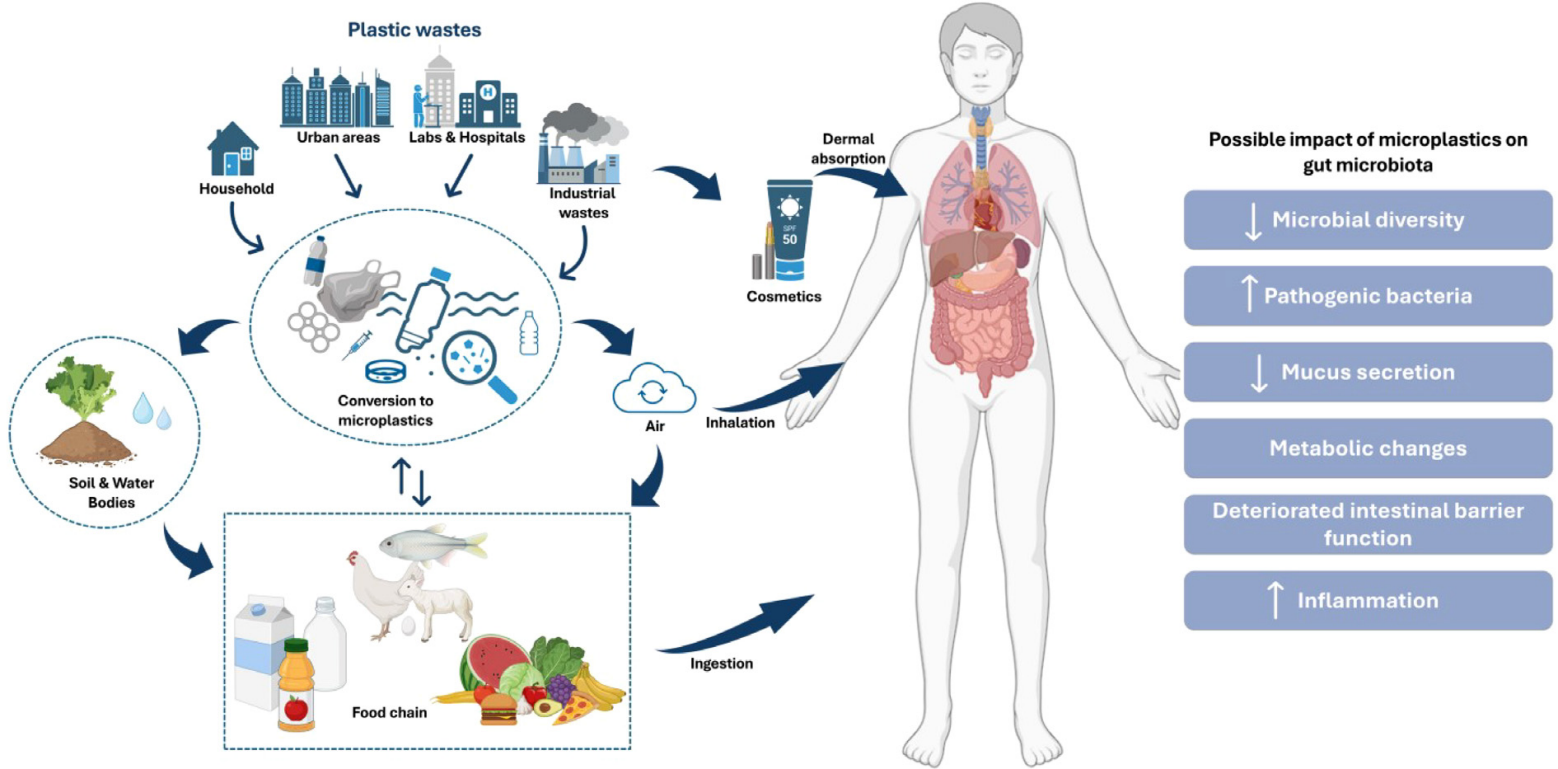
Pathways of microplastic (MP) exposure and its impact on human gut microbiota. The figure illustrates the sources of plastic waste and their transformation into MPs which enter the human body through ingestion, inhalation, and dermal absorption. It also depicts the possible impacts of MPs on gut microbiota, including reduced microbial diversity, increased pathogenic bacteria, altered mucus secretion, metabolic changes, impaired intestinal barrier function, and increased inflammation (Covello et al., 2024; Demarquoy, 2024).

The impact of MPs on human health has garnered much attention in recent years, particularly their effects on the gut microbiome and the subsequent systemic consequences. The presence of MPs in the GI tract can lead to significant microbial disruptions (gut dysbiosis, a state of microbial imbalance), including the reduction of commensal bacteria, which play crucial roles in nutrient provision and pathogen defense (**Figure 2**). For instance, ingestion of polyethylene may increase the abundance of Firmicutes and Bacteroidetes (Campanale et al., 2020; Emenike et al., 2023). MP contamination in Indonesian coastal and highland populations correlated with specific bacterial taxa (*Roseburia, Clostridium*, and *Prevotella*) and plastic-degrading enzyme genes in the gut microbiome (Nugrahapraja et al., 2022). A study explored the effects of chronic polyethylene (PE) microplastic exposure on infant gut microbiota and barrier integrity. PE MPs increased the abundance of harmful pathobionts, including Dethiosulfovibrionaceae and Enterobacteriaceae, while reducing butyrate production in the gut (Fournier et al., 2023). There are few studies on how MP affects the microbiota in human guts to date. Research using non-human animal models has contributed much of our knowledge regarding the effects of MP consumption on gut microbiota architecture. MPs ingested by wild seabirds, such as northern fulmars and Cory’s shearwaters, significantly altered their gut microbiomes. Increased MP presence correlated with decreased commensal microbiota and increased pathogens, antibiotic-resistant, and plastic-degrading microbes (Fackelmann et al., 2023). A systematic review analyzed 28 preclinical studies on the impact of MPs on intestinal microbiota and mucosa of zebrafish and mice as model organisms. The study found MPs to trigger dysbiosis, increasing Firmicutes, Proteobacteria, and Chlamydia, while reducing Bacteroidetes (Souza-Silva et al., 2022). A 16S rRNA sequencing and metabolomics-based study investigated how polystyrene microplastic exposure affects the gut microbiota of C57BL/6 model mice. MP exposure significantly altered gut microbiota composition, diversity, and functional pathways, leading to changes in metabolite profiles related to cholesterol, bile acids, and short-chain fatty acids (Tu et al., 2023). Another study investigated how nylon MPs affect *Chironomus sancticaroli* larvae. While bacterial alpha diversity remained stable, the presence of MPs influenced specific taxa, notably Alphaproteobacteria, Betaproteobacteria, Actinobacteria, and Gammaproteobacteria, altering microbiome structure (Palacio-Cortés et al., 2022). Similarly, MP exposure (at concentrations of 0.125 μg, 0.25 μg, or 0.5 μg per diet for 5 days) did not significantly affect the survival of silkworms (*Bombyx mori*) but disrupted gut microbiota diversity and signaling pathways related to development and cocoon production (Zhang et al., 2024). Microplastics (MPs), particularly polyethylene terephthalate (PET), can undergo biotransformation in the GI tract, as simulated using a static and dynamic GI model. The simulation revealed MPs altering human colonic microbiota composition and potentially forming biofilms (Tamargo et al., 2022).

MPs not only alter the gut microbiome but also affect intestinal structure and function. They can increase intestinal permeability, trigger inflammatory responses, and induce oxidative stress, all of which contribute to metabolic changes and impact liver function and overall metabolism. Furthermore, MPs can act as carriers for pathogens and other pollutants, further complicating their adverse effects. As these particles accumulate in the GI tract, they disturb the delicate balance of microbial communities, exacerbating dysbiosis. This microbial disruption has been linked to various chronic diseases, including obesity, cancer, inflammatory bowel disease, and autism (Tamargo et al., 2022). The altered microbiome compromises the gut’s critical role as a barrier and regulatory organ, setting the stage for systemic inflammation and chronic diseases that affect the entire body. Research has shown that MPs can translocate from the gut to vital organs such as the liver, kidneys, and brain, where they may cause local damage and systemic dysfunction. Additionally, MPs have been detected in breast milk, testicles, and even the heart, highlighting their pervasive nature and potential to induce wide-ranging health effects, including energy imbalance, metabolic disorders, and oxidative stress (Wang et al., 2024). Prolonged exposure to MPs has also been implicated in the disruption of the blood-brain barrier, posing a risk to neurological health (Ibrahim et al., 2021). The pervasive presence of MPs in the environment and their ability to infiltrate the human body underscore the urgent need for further research to fully understand their health implications.

### 4.2 Microplastic exposure, inflammatory responses and gut permeability

**Figure 3:**
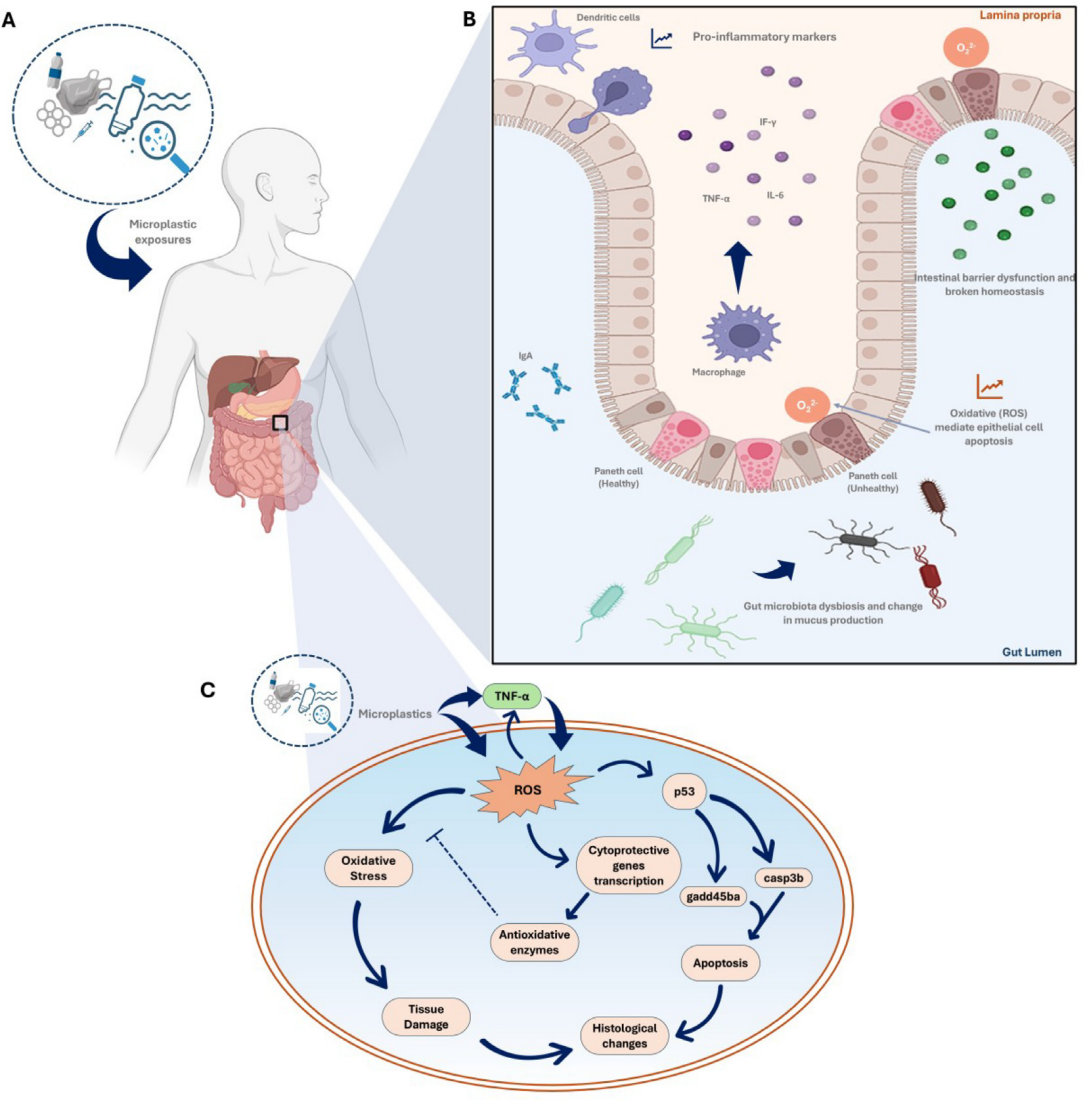
The complex interactions between microplastics (MPs) and the human gut, emphasizing the inflammatory response, alterations in gut microbiota, and potential downstream effects on health through oxidative stress and tissue damage. **(A)** Microplastic exposure (the entry of MPs into the human body); **(B)** MP-induced inflammatory response in the gut focusing on the gut’s immune response to MP-exposure. It shows how dendritic cells and macrophages recognize MPs and release pro-inflammatory cytokines (like TNF-α and IL-6). The presence of these cytokines leads to increased oxidative stress, and disrupts the intestinal barrier function. Healthy Paneth cells are involved in maintaining gut health, but MP-exposure can impair their function. **(C)** Pathways of oxidative stress and damage outlining the cellular pathways activated by MP-induced inflammation (Bahadur et al., 2023; Wallaeys et al., 2023; Sofield et al., 2024).

The gut is home to a significant portion of the body’s immune cells, and its integrity is crucial for maintaining immune homeostasis. The gut microbiota plays a critical role in promoting immune system maturation. For example, *Lactobacillus* and *Bifidobacterium* synthesize folic acid, which can enhance DNA methylation and mRNA N6-methyladenosine (m6A) in intestinal cells, while anaerobic bacteria, Clostridial clusters, and eubacteria can induce butyrate-modified histone acylation, promoting gut development and immune homeostasis (Zhao et al., 2023a). The continuous interaction between the gut microbiota and intestinal epithelium leads to constant immune signaling, which is essential for maintaining intestinal homeostasis. When this process is impaired, it can result in inflammation and infection (Wiertsema et al., 2021). MP-induced gut dysbiosis may trigger inflammatory responses and increase gut permeability, a condition commonly referred to as ‘leaky gut’. This occurs when the tight junctions between epithelial cells in the gut lining become compromised, allowing harmful substances, including toxins, pathogens, and undigested food particles, to pass from the gut into the bloodstream. The compromised permeability of the epithelial cells lining the GI tract facilitates the passage of commensal bacteria, their metabolic products, and pro-inflammatory antigens to move from the gut lumen to the bloodstream, triggering an inflammatory response from local and systemic immune cells (**Figure 3**). Over time, chronic gut dysbiosis and the translocation of bacteria and their metabolic products across the mucosal barrier can increase the prevalence of various diseases (Yoo et al., 2020).

The association between leaky gut and autoimmune diseases has been well-documented, with research highlighting that the leakage of pathogens into the systemic circulation can lead to autoimmunity, where the immune system mistakenly attacks the body’s own tissues (Fasano, 2011). The presence of MPs exacerbates this condition through several mechanisms. MPs can physically irritate the gut lining, with the sharp edges of these particles causing microabrasions that disrupt the integrity of the gut barrier. Additionally, MPs can carry and release harmful chemicals, such as heavy metals and persistent organic pollutants (POPs), which further damage the gut lining and contribute to oxidative stress and inflammation (Lee et al., 2023). These toxic substances can induce the release of pro-inflammatory cytokines, including TNF-α, IL-6, and IL-1β, perpetuating a state of chronic inflammation within the gut.

Microbial products like lipopolysaccharides (LPS) entering the bloodstream can invoke systemic immune responses and chronic inflammation, a risk factor for autoimmune diseases such as rheumatoid arthritis, lupus, and inflammatory bowel disease (IBD) (Schwarzfischer and Rogler, 2022). Dysbiosis can impair the gut’s ability to produce regulatory T cells (Tregs), essential for maintaining immune tolerance. An imbalance between Tregs and Th17 cells may exacerbate conditions like multiple sclerosis (MS) and other autoimmune disorders (Shim et al., 2023). Treg dysfunction is linked to several autoimmune disorders, including type 1 diabetes, rheumatoid arthritis, and systemic lupus erythematosus (SLE), with mutations in the Treg transcription factor FOXP3 further driving autoimmune disease development (Oparaugo et al., 2023). The altered microbial composition may affect the development and function of other immune cells, such as dendritic cells and macrophages, leading to compromised immune responses (Shao et al., 2023). Moreover, MPs act as vectors for harmful pollutants, capable of transporting contaminants such as pesticides, polychlorinated biphenyls (PCBs), and heavy metals into the food chain and ultimately into the human body (Zhang et al., 2020). The ingestion of MPs increases exposure to these chemicals, leading to potential poisoning and other adverse health effects. MPs can also stimulate the release of endocrine disruptors, interfering with hormone signaling and potentially leading to reproductive and developmental issues. Studies suggest that micro- and nano-plastics (MNPs) may possess endocrine-disrupting properties, further implicating them in reproductive and developmental health concerns (Kumar et al., 2022).

### 4.3 Microplastic induced gut dysbiosis and links to chronic liver diseases

The systemic effects of MP exposure are far-reaching, as these particles can migrate to various organs, including the liver, kidneys, and brain, where they may alter metabolic pathways and contribute to carcinogenesis through mechanisms such as DNA fragmentation, oxidative stress, and genomic alterations. MP accumulation in these organs raises significant concerns about potential long-term health effects related to chronic exposure (Han et al., 2019). The consequences of intestinal dysbiosis can be severe, including the onset of endotoxemia, an inflammatory response, and further impairment of the intestinal barrier. Such injuries are associated with the development of health disorders like nonalcoholic fatty liver disease (NAFLD) (Auguet et al., 2022).

The ‘gut-liver axis’ i.e., the interplay between the bowel, liver, and gut microbiota offers a new perspective on understanding the possible toxic effects of MPs in human health. The liver is the primary organ for xenobiotic (including MPs) detoxification and excretion. Studies in zebrafish and mouse models have shown that MP-exposure can increase oxidative stress and promote lipogenic and inflammatory gene expression in liver (Li et al., 2024). Male mice orally exposed to green fluorescent MPs (even at 0.1 mg/L) for two months showed DNA damage in both the nucleus and mitochondria. This triggered the cGAS–STING pathway (an innate immune system), leading to liver fibrosis (Shen et al., 2022). Male mice exposed to polystyrene MPs of sizes 0.5 and 50 μm for 5 weeks showed decreased body, liver, and lipid weights. Hepatic triglyceride (TG) and total cholesterol levels decreased in both MP-treated groups. Although the transcription of triglyceride synthesis-related genes (Dgat1, Dgat2, and Gpat) remained unchanged in the liver, they showed significant reductions in epididymal fat. MP exposure also affected fatty acid transport genes (Fatp2 and Fat), while increased pyruvate kinase mRNA levels (Lu et al., 2018). In a similar mouse model study, oral administration of 0.5 μm sized polystyrene MPs for one month at 0.5 mg/day increased liver weight and function parameters, led to the infiltration of NK cells and macrophages in the liver (Zhao et al., 2021). In liver, MPs trigger various cell death programs, including apoptosis (via the p53/Bcl-2/Bax pathway), pyroptosis, and ferroptosis (via NLRP3/ASC pathways). Male mice exposed to 5 μm fluorescent polypropylene MPs for a month showed liver damage, including disrupted mitochondrial cristae, elevated liver enzyme activity, pyroptosis, oxidative damage, and lipid peroxidation, indicating significant hepatic stress and injury (Mu et al., 2022).

The risk of MP exposure is evident as six types of MP polymers have been identified exclusively in the cirrhotic human liver samples (Horvatits et al., 2022). Human liver organoids exposed to even 1 μm polystyrene MPs experienced hepatotoxicity, lipotoxicity, oxidative stress, and inflammation. The exposure increased apoptotic cells, liver damage markers, and expression of genes linked to liver steatosis and fibrosis (Cheng et al., 2022). An *in vitro* study using human hepatocytes (Hep G2) found that polystyrene MPs (100 μg/mL) reduced cell proliferation and altered gene expression for hepatocyte glycolysis and antioxidant enzymes, potentially disrupting metabolism in the liver (Ali et al., 2024). In human liver organoids, MPs upregulate lipid metabolism, insulin signaling, and mitochondrial function genes (especially, oxidative stress linked CYP2E1). It can activate proinflammatory Kupffer cells to modulate inflammation (Cheng et al., 2022).

### 4.4 MP-Induced gut dysbiosis, the gut-heart axis and cardiovascular health

The gut-heart axis, a dynamic connection between gut health and cardiovascular function, is mediated by gut microbiota, immune responses, and metabolic processes. A healthy gut microbiome maintains gut lining integrity, immune regulation, and produces metabolites like short-chain fatty acids (SCFAs) that influence heart function (Blaak et al., 2020). MP-induced dysbiosis disrupts this balance, allowing inflammatory molecules and bacterial endotoxins to enter circulation, fueling chronic inflammation linked to CVDs like atherosclerosis, hypertension, and vascular dysfunction (Hrncir, 2022). Dysbiosis also alters cholesterol metabolism and contributes to coronary artery disease (CAD) development, as evidenced by associations between gut microbiome composition and metabolic disorders (Trøseid et al., 2020). Dysbiosis can compromise the intestinal mucosa barrier, allowing harmful metabolites like Trimethylamine N-oxide (TMAO) to enter the bloodstream, reach the liver, and contribute to chronic inflammation that damages the cardiovascular system (Canyelles et al., 2023). The systemic inflammation caused by increased gut permeability and MP-induced dysbiosis can exacerbate plaque formation in arteries, heightening the risk of heart attacks and strokes (Shen et al., 2021). The ‘leaky gut’ allows bacterial products, such as lipopolysaccharides (LPS) from Gram-negative bacteria, to enter the bloodstream, resulting in a pro-inflammatory state linked to cardiovascular diseases (CVD) (Candelli et al., 2021). When the gut barrier fails, LPS activates toll-like receptors (TLRs) on immune cells, triggering inflammatory responses. Elevated endotoxin levels in decompensated heart failure patients are associated with inflammation and vascular dysfunction, and circulating LPS can predict major adverse cardiovascular events (MACE) (Candelli et al., 2021).

A prospective observational study examined the presence of MPs in carotid artery plaques from patients undergoing carotid endarterectomy for asymptomatic carotid artery disease. The study detected polyethylene in 58.4% of patients’ plaques and polyvinyl chloride in 12.1%. The study found that patients with detectable MPs in their plaques had a significantly higher risk of major cardiovascular events (myocardial infarction, stroke, or death) compared to those without MPs, with a hazard ratio of 4.53. These findings suggest a strong link between the presence of MPs in atheromas and increased cardiovascular risk (Marfella et al., 2024). Another cross-sectional study investigated the presence of MPs in the blood of patients with chest pain and their potential link to acute coronary syndrome (ACS). The study reported that ACS patients, particularly those with acute myocardial infarction, had higher concentrations of MPs compared to controls and those with unstable angina. Elevated MP levels were associated with increased inflammatory markers (IL-6, IL-12p70) and higher counts of B lymphocytes and natural killer cells. These findings strongly prove a connection between MPs, vascular pathology, and immune-inflammatory responses in cardiovascular diseases, highlighting the need for further research (Yang et al., 2024).

### 4.5 Microplastic induced gut dysbiosis and renal health

The gut-kidney axis highlights the interdependent relationship between gut health and kidney function, influenced by metabolic, microbial, and immune pathways. MP-exposure disrupts this axis, leading to potential kidney dysfunction by causing gut dysbiosis, which weakens the intestinal barrier and increases permeability. This allows harmful substances, including bacterial toxins and inflammatory molecules, to enter the bloodstream, burdening the kidneys responsible for waste filtration (Yang et al., 2018). This disruption activates immune responses, leading to systemic inflammation that drives kidney damage, fibrosis, and oxidative stress, exacerbating chronic kidney disease (CKD). The ubiquitous presence of MPs, including pigmented MP fragments ranging from 4 to 15 μm in size, has been confirmed in human urine through Raman microspectroscopy, indicating that they can traverse the GI tract and be excreted from the body (Pironti et al., 2022). A study examined the effects of polystyrene MP exposure on the kidneys of mice, using MPs of three different diameters (80 nm, 0.5 µm, and 5 µm). The results showed that MPs caused varying levels of kidney damage, inducing inflammation, oxidative stress, cell apoptosis, and ultimately leading to kidney fibrosis. Transcriptome analysis revealed that chronic MP (80 nm) exposure induced immune modulation and immune feedback in the murine kidney (Xiong et al., 2023). Another study investigated the combined effects of polystyrene MPs and cadmium (Cd) on kidney health by exposing mice to 5 μm MPs (10 mg/L) and CdCl_2_ (50 mg/L) for three months. The results showed that MPs worsened Cd-induced kidney damage by increasing oxidative stress, autophagy, apoptosis, and fibrosis. These findings highlight the harmful effects of MPs in amplifying heavy metal toxicity in the kidneys, offering new insights into their combined impact on kidney injury (Zou et al., 2022).

### 4.6 Microplastic exposure, gut-brain axis and neurological implications

The inflammatory response induced by MP exposure may also affect the gut-brain axis, a complex bidirectional communication network between the GI system and the central nervous system (Sofield et al., 2024). The GBA utilizes immune, endocrine, neural, and humoral connections to maintain communication. Dysbiosis can disrupt these functions, leading to neuroinflammation, which is implicated in neurological and psychiatric conditions such as anxiety, depression, and cognitive decline (Anand et al., 2022). For example, dysbiosis may reduce the production of short-chain fatty acids (SCFAs), which are crucial for brain health, leading to an increase in pro-inflammatory cytokines that can negatively impact brain function (Silva et al., 2020). C57BL/6 mice exposed to 5 μm polystyrene MPs for 28 days exhibited anxiety-like behavior, hippocampal inflammation as assessed by behavioral tests. The interplay between the gut microbiome, brain, and immune system is complex and multifaceted. The gut microbiome influences brain chemistry, stress response, and memory function, while also impacting immune system regulation. For instance, the gut microbiome affects the activity of CD8+ T cells, which are crucial for immune defense and tumor surveillance, with specific probiotic species influencing their efficacy (Petakh et al., 2024). Signals from gut microbes can stimulate cells in the periphery to travel to the brain, and gut bacteria can bind to receptors in the vagus nerve, affecting central nervous system inflammation (Bostick et al., 2022). This interconnectedness suggests that MP exposure not only contributes to chronic diseases and immune disorders but also amplifies their severity and complexity. However, it remains unclear to what extent the damage is directly caused by MP accumulation within cells and tissues compared to functional changes originating in the gut and disruption of the gut-brain axis. A recent study has analyzed MP accumulation in human kidneys, livers, and brains using autopsy samples from 2016 and 2024. The results showed that MP concentrations were highest in the brain compared to the liver and kidneys, with a significant increase in MP levels across all organs from 2016 to 2024. Polyethylene was the most abundant polymer, particularly in the brain. The findings indicate that MPs selectively accumulate in the brain and that their presence is increasing over time (Campen et al., 2024). A similar study has reported the presence of MPs in olfactory bulb tissues obtained from autopsies in São Paulo, Brazil. MPs were detected in the olfactory bulbs of 8 out of 15 individuals, with a total of 16 synthetic polymer particles and fibers identified. The most common polymer found was polypropylene (43.8%) with particle sizes ranging from 5.5 to 26.4 μm. These findings suggest that MPs may also translocate to the brain via the olfactory bulb, raising concerns about the potential neurotoxic effects and the ability of MPs to bypass the blood-brain barrier (Amato-Lourenço et al., 2024).

## 5 Conclusion

Mitigating the effects of microplastics (MPs) on human health requires a multifaceted strategy. Governments and industries must collaborate to regulate plastic production, enhance waste management, and develop alternatives, including biodegradable materials. Stricter regulations on plastic disposal and recycling are essential, alongside monitoring MP levels in water, air, and food. Innovations in filtration technologies and materials, public education, and dietary choices are crucial. Reducing MP exposure through minimal plastic packaging, avoiding single-use plastics, filtering drinking water, and improving air quality are important preventive measures. Standardized monitoring methods, better waste treatment technologies, and stricter controls on plastic production are essential steps forward.

In recent times, fecal microbiota transplantation (FMT) has shown promise as a therapeutic avenue for various conditions, including gastrointestinal, cardiovascular, inflammatory, autoimmune diseases, metabolic disorders, etc. by altering the gut microbiome. FMT involves transferring stool from a healthy donor to a patient, with the goal of treating illness (Soo et al., 2020). A study has shown that transplanting fecal microbiota from healthy human donors in *Caenorhabditis elegans* mitigates nano-plastics toxicity by activating the PMK-1/SKN-1 pathway, increasing intracellular anti-oxidative glutathione production (Chu et al., 2021). Another recent study has investigated the combined toxic effects of doxycycline (Dox) and polystyrene MPs on mice. The results showed that their co-exposure disrupted gut microbiota homeostasis, leading to brain lesions and inflammation, and impaired learning and memory through the gut-brain axis. Importantly, FMT reversed neurological impairments from combined exposure, restoring cognitive functions (Sun et al., 2024). Studies demonstrate that administering probiotic *Akkermansia muciniphila* can enhance gut barrier function by decreasing intestinal permeability and lowering LPS levels, potentially reducing aortic atherosclerosis (Mo et al., 2024; Zhao et al., 2024). These studies suggest FMT as a promising strategy to mitigate their combined effects. In the near future, identifying and characterizing super-donor gut microbiomes could lead to the creation of stool microbiota banks, supporting the treatment of microplastic-induced health conditions. Additionally, promoting gut health with a fiber-rich, prebiotic, and probiotic diet, along with regular exercise and stress management, can help counteract MP-induced gut dysbiosis. More research is needed to explore bioremediation techniques, understand chronic health effects like cancer and metabolic disorders, and investigate the impact of MPs on cellular processes, sensitive groups, and ecosystems.
